# Artificial intelligence-assisted delineation for postoperative radiotherapy in patients with lung cancer: a prospective, multi-center, cohort study

**DOI:** 10.3389/fonc.2024.1388297

**Published:** 2024-10-22

**Authors:** Ziming Han, Yu Wang, Wenqing Wang, Tao Zhang, Jianyang Wang, Xiangyu Ma, Kuo Men, Anhui Shi, Yuyan Gao, Nan Bi

**Affiliations:** ^1^ National Cancer Center/National Clinical Research Center for Cancer/Cancer Hospital, Chinese Academy of Medical Sciences and Peking Union Medical College, Beijing, China; ^2^ Department of Radiation Oncology, Peking University Cancer Hospital & Institute, Beijing, China; ^3^ Department of Radiation Therapy, Beijing Luhe Hospital Affiliated to Capital Medical University, Beijing, China

**Keywords:** lung cancer, postoperative radiotherapy, artificial intelligence, automatic contour, target delineation

## Abstract

**Background:**

Postoperative radiotherapy (PORT) is an important treatment for lung cancer patients with poor prognostic features, but accurate delineation of the clinical target volume (CTV) and organs at risk (OARs) is challenging and time-consuming. Recently, deep learning-based artificial intelligent (AI) algorithms have shown promise in automating this process.

**Objective:**

To evaluate the clinical utility of a deep learning-based auto-segmentation model for AI-assisted delineating CTV and OARs in patients undergoing PORT, and to compare its accuracy and efficiency with manual delineation by radiation oncology residents from different levels of medical institutions.

**Methods:**

We previously developed an AI auto-segmentation model in 664 patients and validated its contouring performance in 149 patients. In this multi-center, validation trial, we prospectively involved 55 patients and compared the accuracy and efficiency of 3 contouring methods: (i) unmodified AI auto-segmentation, (ii) fully manual delineation by junior radiation oncology residents from different medical centers, and (iii) manual modifications based on AI segmentation model (AI-assisted delineation). The ground truth of CTV and OARs was delineated by 3 senior radiation oncologists. Contouring accuracy was evaluated by Dice similarity coefficient (DSC), Hausdorff distance (HD), and mean distance of agreement (MDA). Inter-observer consistency was assessed by volume and coefficient of variation (CV).

**Results:**

AI-assisted delineation achieved significantly higher accuracy compared to unmodified AI auto-contouring and fully manual delineation by radiation oncologists, with median HD, MDA, and DCS values of 20.03 vs. 21.55 mm, 2.57 vs. 3.06 mm, 0.745 vs. 0.703 (all P<0.05) for CTV, respectively. The results of OARs contours were similar. CV for OARs was reduced by approximately 50%. In addition to better contouring accuracy, the AI-assisted delineation significantly decreased the consuming time and improved the efficiency.

**Conclusion:**

AI-assisted CTV and OARs delineation for PORT significantly improves the accuracy and efficiency in the real-world setting, compared with pure AI auto-segmentation or fully manual delineation by junior oncologists. AI-assisted approach has promising clinical potential to enhance the quality of radiotherapy planning and further improve treatment outcomes of patients with lung cancer.

## Introduction

Lung cancer is one of the most common types of cancer worldwide, and postoperative radiotherapy (PORT) remains an important intervention for this disease (1–4). Accurate delineation of the clinical target volume (CTV) and organs at risk (OARs) is essential for optimal radiation treatment planning and delivery. However, manual delineation of these structures is time-consuming and varies widely among radiation oncologists (5). Hence, the promotion of accurate and efficient methods to automate the process of radiation target delineations in clinical scenarios is highly desirable (6, 7). Currently, artificial intelligence (AI) technology not only develops rapidly, but also has found its widespread applications in clinical medicine, especially radiation oncology (8–10). The advent of deep learning algorithms has revolutionized the medical data processing and image auto-segmentation, offering novel opportunities to improve accuracy and reduce variability during radiation planning (5). Deep learning-based systems have shown great potential in automating the segmentation of medical images, without the need for explicit feature extraction or segmentation rules, and greatly facilitated the promotion of intelligent oncology (11).

Previous studies have demonstrated the promising potential of deep learning-based auto-segmentation models in improving the accuracy and efficiency of auto-delineating target volumes in non-small cell lung cancer (NSCLC) patients undergoing radiotherapy, which reduced the inter-practitioner variabilities and the time cost and allowed for rapid treatment planning and adaptive replanning for the benefit of patients (12–15). Despite a high level of acceptance among physicians for the adoption of AI contouring technology in the real-world setting (16–18), there is a lack of high-level evidence or prospective trials directly validating the clinical usefulness of AI-assisted models, which limits the practical application of AI-associated automated models in the field of radiotherapy. Validation of AI models in multi-centers and comparison with pure AI segmentation tools and completely manual rendering can increase clinical evidence and break the dilemma of difficulty in popularizing and convincing AI models.

In this prospective, multi-center, validation study, we evaluated the clinical utility of AI-based contouring model, which was developed and validated in our previous research using the deep dilated convolutional neural network (DDCNN) (13, 19), in delineating CTV and crucial OARs for patients with lung cancer receiving PORT. We compared three delineation strategies: (i) unmodified pure AI auto-segmentation, (ii) fully manual contours by junior radiation oncology residents, and (iii) independently manual modifications based on AI segmentation (AI-assisted delineation), aiming to identify the optimal delineation strategy and determine the value of AI technology in clinical practice. We hypothesize that the AI-assisted technique would outperform the other two methods and further improve the efficiency and accuracy of pure AI or fully manual contours. This research will provide important insights into the clinical benefit and potential limitations of using deep learning algorithms in radiotherapy for patients with lung cancer.

## Materials and methods

### Study design and eligible criteria

**Figure 1:**
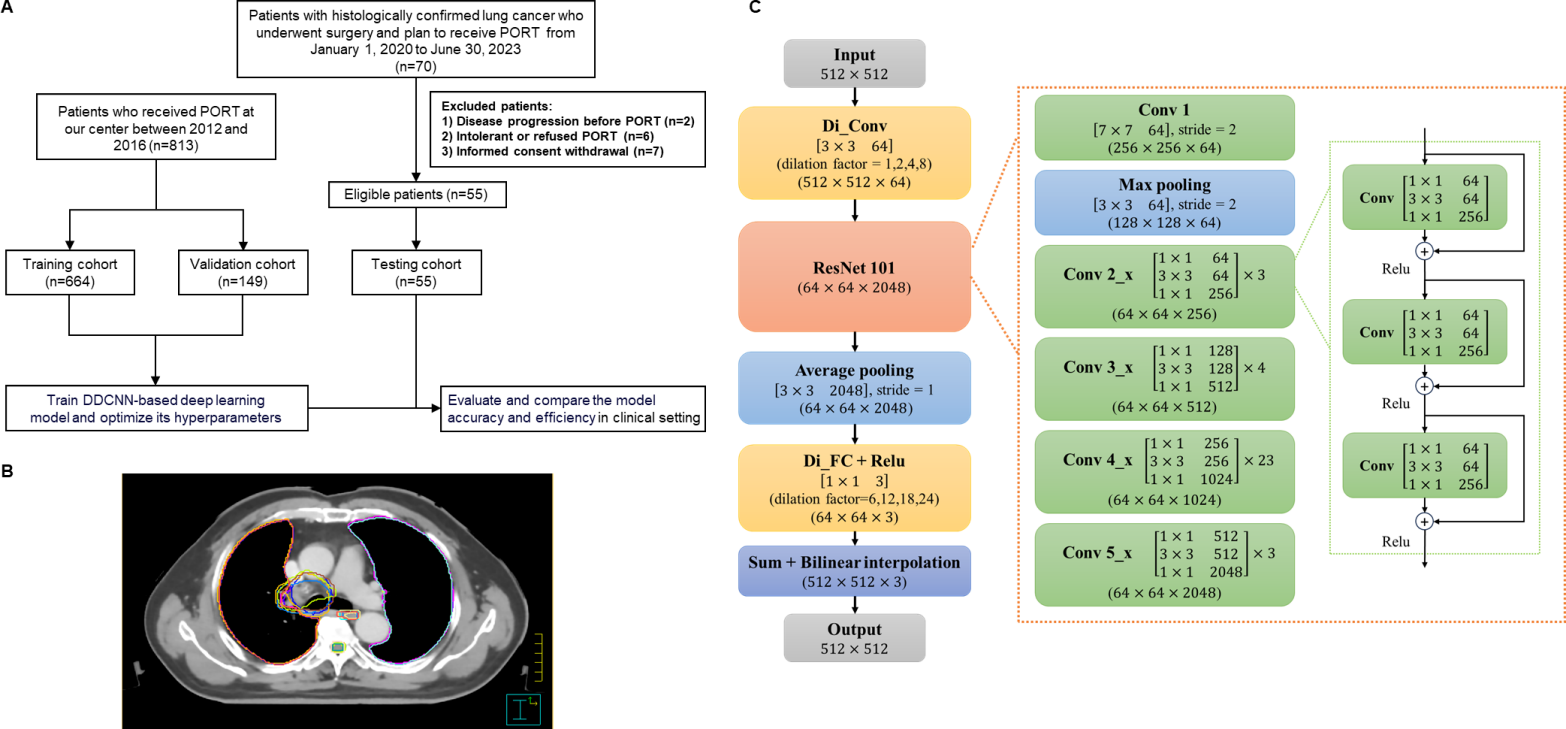
Study overview. **(A)** Flow diagram. **(B)** Examples of CTV and OAR delineated by different radiation oncologists using two methods. **(C)** Architecture of DD-ResNet-101 and DDCNN. PORT, postoperative radiotherapy; DDCNN, deep dilated convolutional neural network.

In this prospective, multi-center study, 70 patients with lung cancer undergoing surgery and suitable for PORT were consecutively enrolled from 3 different medical centers (Cancer Hospital of Chinese Academy of Medical Sciences [CAMS], Beijing Cancer Hospital and Institute [BJCH], and Beijing Luhe Hospital of Capital Medical University [LH]) from January 2020 to June 2023. Patients with pathologically confirmed lung cancer after surgery planning to receive PORT were included. Patients who developed disease progression or failed to meet the indications for PORT for any reason were excluded. A total of 55 eligible patients were identified as the testing set and included into the final analysis (**Figure 1A**). All participants adopted four-dimension computerized tomography (CT) simulation scans for radiotherapy target delineation. Patients were placed in a supine position, with their hands crossed over their heads and fixed with cervical pleura or body membrane. The scanning thickness was 3 to 5 mm, and 55 sets of localization images were finally collected. CT images from different medical centers are transmitted into the Medical Digital Imaging and Communication (DICOM) format.

### Study procedures and contouring methods

As shown in **Figure 1**, the AI automatic delineation model was developed using the DDCNN deep learning algorithm based on the testing and validation set. Specifically, it was trained on a retrospective set of 664 patients with lung cancer receiving PORT with manually delineated CTV and OARs by 3 senior radiation oncologists (the training set) (19), and was independently validated and optimized using the external dataset of 149 patients diagnosed with lung cancer (the validation set) between 2012 and 2016 at Cancer Hospital of CAMS (8, 19). Subsequently, this multi-center trial, which prospectively involved 55 patients and constituted the testing set, sought to demonstrate the contouring efficiency and accuracy of DDCNN AI auto-segmentation model in the clinical setting.

Each set of images from 55 eligible patients (the testing set) was contoured in 3 different methods: 1) pure AI automatic segmentation, which generated 55 sets of target volumes; 2) fully manual contouring by 3 radiation oncology residents from 3 institutions, which totally generated 165 sets of targets; 3) AI-assisted delineation, that is, independently manual modifications from 3 radiation oncology residents based on AI auto-segmentation, which generated 165 sets of contours (**Figure 1B**). The order of the three methods was randomized and balanced across patients to avoid potential biases. We evaluated and compared the accuracy and efficiency of these 3 delineation methods in the testing dataset.

### Ground truth of CTV and OARs delineation

The standard ground truth (GT) for CTV and OARs contours was established by 3 senior radiation oncologists from 3 different institutions, all of whom have extensive experience in radiotherapy for thoracic cancer (N.B., A.S., and Y.G., with more than 10 years of working experience in this field). in conformity to the protocol of a phase III randomized controlled trial focusing on PORT in lung cancer (NCT00880971) (2). To ensure unbiased evaluation, they worked independently on the same set of PORT images without knowledge of each other’s contours. The majority voting after careful consultation and discussion was used to generate the GT, and it was used to train the AI auto-segmentation model and serve as the reference gold standard for evaluating the performance of both automatic and manual delineations.

### Deep learning algorithm for AI segmentation model

The AI auto-segmentation method, based on DDCNN deep learning algorithm, was trained on a retrospective dataset of 664 lung cancer patients undergoing PORT with manually delineated CTV and OARs (the training set) (19). The model was developed in our previous work and further optimized using a combination of cross-validation. Overall, the training set, including CT images and manual segmentation labels, was used to adjust the parameters and establish a robust DDCNN-based auto-segmentation model. The validation set was used to evaluate the performance of this AI model, and further improve its contouring effectiveness.

Moreover, **Figure 1C** illustrates the detailed architectures of deep dilated residual network (DD-ResNet), a robust deep learning algorithm to automatically segment CTV and OAR structures. We established a 4-stream dilated convolutional module and integrated it with the ResNet-101 network. DD-ResNet could adeptly capture original context information, leveraging different dilated factors to achieve large receptive fields that could extract multi-scale contextual features. The resulting multi-scale feature maps were then added to a specific feature number and fed forward to the ResNet-101. ResNet-101 is a fully convolutional network architecture, and at large extracts low-level, mid-level, and high-level visual features, which in turn are utilized in the pixel-level classification task. Additionally, optimizing deep convolutional networks can be challenging due to the vanishing gradients, which adversely impact semantic segmentation tasks. In response, residual networks (ResNet) add “shortcut connections” to their convolutional layers, which sum with the outputs of those layers to solve the problem of vanishing features.

### Contouring accuracy and consistency assessments

The delineation performances of junior radiation oncology residents were evaluated by various metrics, including the Dice similarity coefficient (DSC), mean distance of agreement (MDA), and the Hausdorff distance (HD), referencing the GT contours (20–22). The spatial overlap between any two contours was calculated using the DSC:


$$DSC\left(\text{A},\text{B}\right)=\frac{2\left|\text{A}\cap \text{B}\right|}{\left|\text{A}\right|+\left|\text{B}\right|}$$

Herein, ***A*** represents the volume of the GT segmentations, while ***B*** represents the volume of an auto-segmented contour. Their intersection (***A*** ∩ ***B***) gives the volume that they have in common. The DSC ranges from 0 to 1, and a DSC value of 0 indicates no overlap while a value of 1 signifies complete overlap between the two contours (the GT and junior resident radiation oncologists). Meanwhile, MDA calculates the average distance between the surfaces of two volumes, with a value of 0 indicating a perfect agreement.

Furthermore, HD is defined as:


HD(A,B)=max(h(A,B),h(B,A))

The value ***h(A,B)*** indicates a point within ***A*** that is farthest from any point of ***B*** and measures the distance from a point to its nearest neighbor in ***B***. The HD value can be calculated as the maximum between ***h(A,B)*** and ***h(B,A)***, which represents the largest degree of mismatch between ***A*** and ***B***. Thus, the degree of overlap between these two volumes increases as the HD value decreases. Both DSC, MDA and HD were calculated using the MIM software (version 6.9.2, Cleveland, OH).

In terms of inter-observer consistency assessments, the coefficient of variation (CV) is calculated as dividing the standard deviation (SD) by the mean CTV volume, which is determined by all observers for each patient and each delineation method. A higher CV value indicates greater variability or lower consistency.

### Statistical analysis

The continuous variables were reported either as mean ± standard deviation (SD) or median with interquartile range (IQR), depending on the normality of the data. Paired t-tests or Wilcoxon signed rank tests were conducted to compare the accuracy and efficiency of different contouring methods on the same set of images from one patient. The t-tests or Mann-Whitney U tests were employed to analyze the differences in the continuous variables between groups. Two-sided P value less than 0.05 was considered statistically significant. SPSS 26.0 was used for data processing and statistical analysis.

## Results

### Patient characteristics

A total of 55 lung cancer patients were finally included in the testing set in this study. The median age of the 55 patients was 57 years old, with the majority of male patients. The main pathological types were adenocarcinoma (60%), squamous cell carcinoma (7%), and small cell lung cancer (20%). The primary tumor sites were upper lobe (52.7%), middle lobe (5.5%), and lower lobe (41.8%). The N2 stage was observed in 81.2% of patients during the surgical phase. The baseline characteristics of the patients are presented in **Table 1**.

**Table 1:** Baseline characteristics of 55 patients.

Characteristics	N (%)
Age, median (range)	57 (28-81)
Gender	
Male	38 (69.1)
Female	17 (30.9)
Tumor location	
Left upper	11 (20.0)
Left lower	10 (18.2)
Right upper	18 (32.7)
Right middle	3 (5.5)
Right lower	13 (23.6)
Pathology	
Squamous cell carcinoma	7 (12.7)
Adenocarcinoma	33 (60.0)
SCLC	11 (20.0)
Other	4 (7.3)
Pathological N category	
N0	6 (11.0)
N1	2 (3.6)
N2	45 (81.8)
N3	2 (3.6)
Pathological T category	
T1	27
T2	21
T3	5
T4	2

SCLC, small cell lung cancer.

### Accuracy evaluation

**Figure 2:**
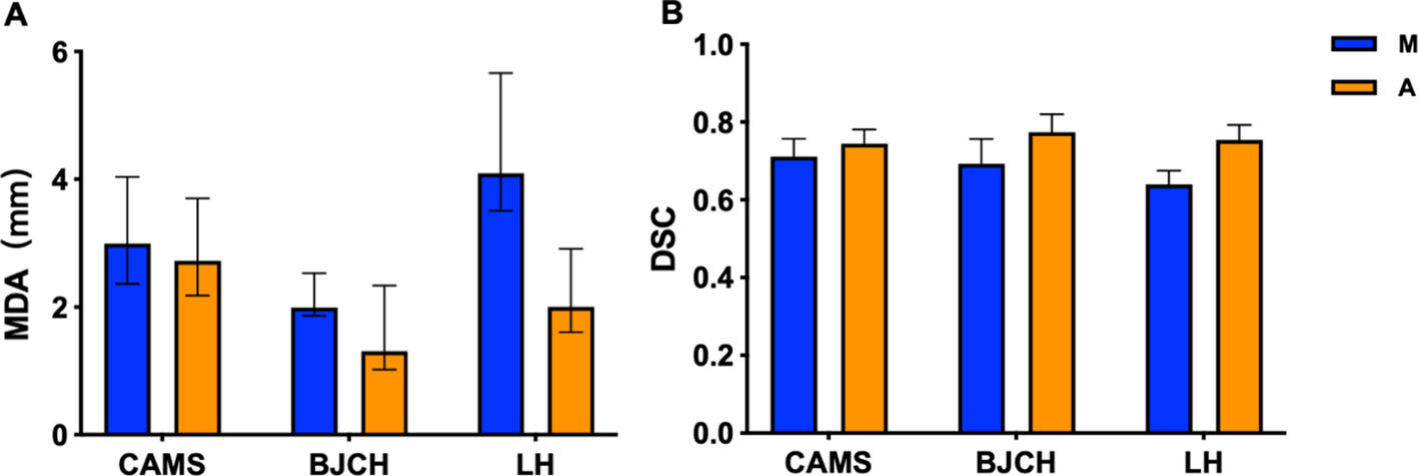
MDA **(A)** and DSC **(B)** of CTV delineated by two methods in different centers. CAMS, Cancer Hospital of Chinese Academy of Medical Sciences; BJCH, Beijing Cancer Hospital and Institute; LH, Beijing Luhe Hospital of Capital Medical University; M (blue), manual contour; A(orange), AI-assisted delineation.

**Figure 3:**
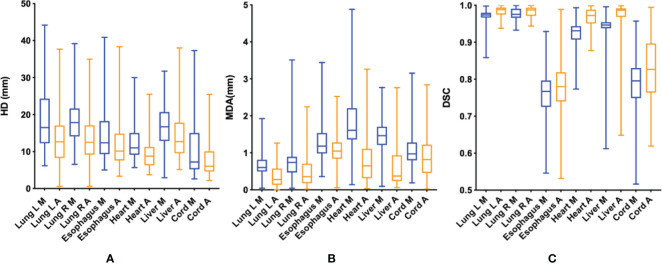
HD **(A)**, MDA **(B)** and DSC **(C)** of OARs delineated by two methods. HD, Hausdorf distance; MDA, mean distance to agreement (mm); DSC, dice coefficient; M (blue), manual contour; A (orange), AI-assisted delineation.

Compared with the GT standard, the AI-assisted CTV delineation had a significantly lower median HD (20.03 [IQR: 14.50, 27.01] vs. 21.55 [IQR: 16.11, 30.51], P< 0.05), and smaller MDA (2.57 [IQR: 2.03, 3.52] vs. 3.06 [IQR: 2.32, 4.11], P< 0.05), higher median DSC (0.745 [IQR: 0.715, 0.784] vs. 0.703 [IQR: 0.656, 0.750], P< 0.05). The accuracy of CTV delineation by two methods in different centers is shown in **Supplementary Table 1**; **Figure 2**. In the manual contour group, the DSC of CAMS group is the highest, and the MDA of BJCH is the lowest. The accuracy of AI-assisted delineation group in the LH group is greatly improved compared with that of the simple manual contour group (DSC: 0.753 VS 0.639), p<0.05). The contouring accuracy of other OARs was consistent with that of CTV. Additionally, the accuracy of the AI-assisted delineation significantly outperformed fully manual contours by junior radiation oncologists, with DSC greater than 0.8, among which the left and right lungs, heart and liver performed better, with DSC greater than 0.9 (all P< 0.05). **Supplementary Table 2**; **Figure 3** presents the parameters of OARs accuracy.

### Consistency assessment

**Figure 4:**
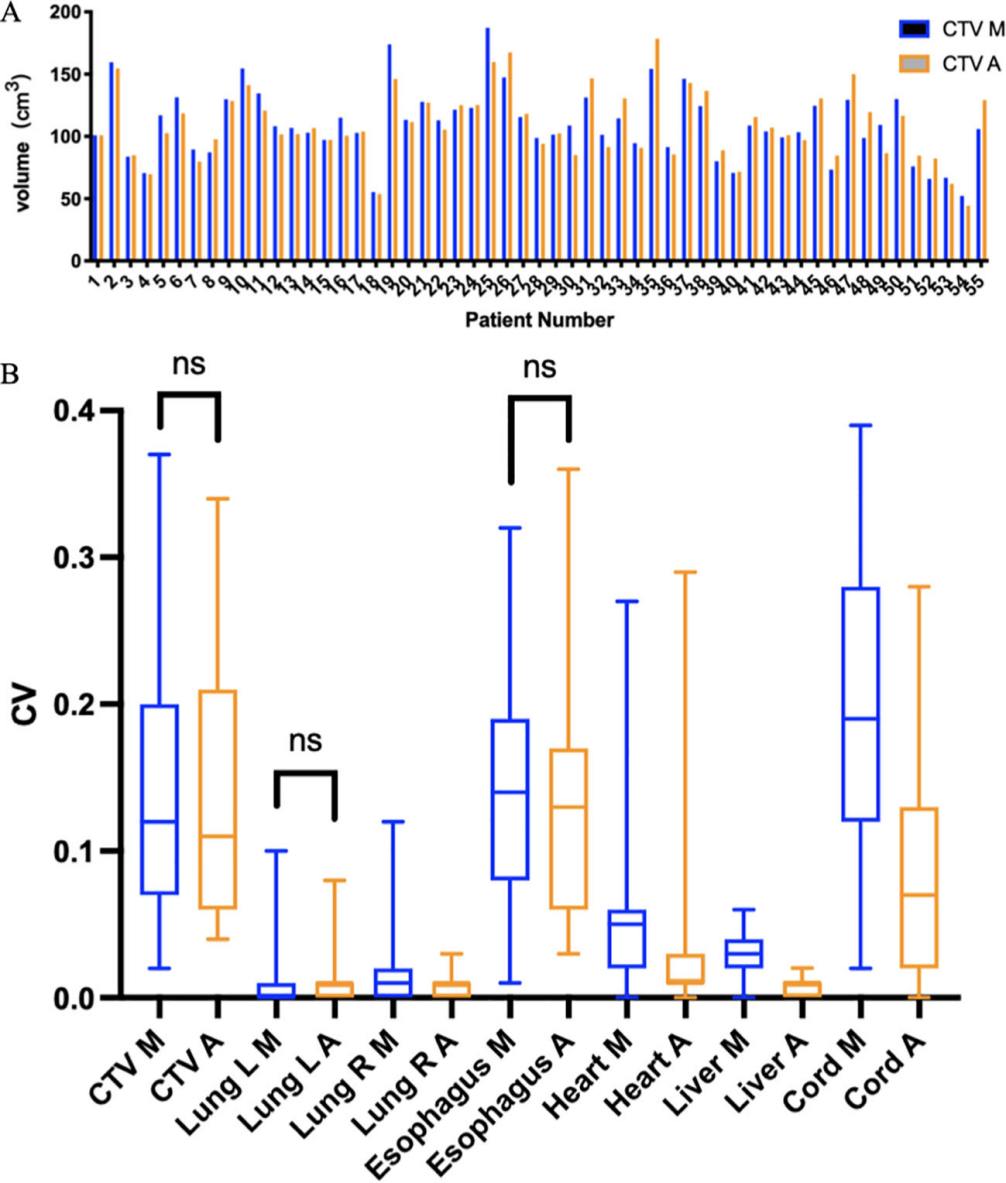
**(A)** CTV volumes delineated using two methods in 55 patients receiving PORT. The blue bar chart (CTV M) represents manual contour, and the orange bar chart (CTV A) represents AI-assisted delineation. **(B)**. Coefficient of variation (CV) of CTV and OARs. M, manual contour; A, AI-assisted delineation. ns, no significance.

As shown in **Figure 4A**, the mean volumes of CTV delineated by AI -assisted and fully manual methods were 109.19 ± 28.18cm^3^ and 109.74cm^3^ ± 28.16cm^3^, respectively (P=0.745), indicating no significant difference in volume between the two methods. In terms of OARs (**Table 2**), the volumes of left lung (1321.75cm^3^ vs. 1362.42cm^3^), right lung (1518.07cm^3^ vs. 1549.46cm^3^), esophagus (32.78cm^3^ vs. 36.30cm^3^), liver (1345.02 cm^3^ vs. 1372.63cm^3^), and spinal cord (40.31cm^3^ vs. 45.52cm^3^) delineated by AI-assisted method were significantly smaller than those by fully manual contours (all P< 0.001). However, no significant difference in the volume of heart was observed. The CV of CTV in the AI-assisted arm was numerically lower than that in the manual delineation arm (0.146 ± 0.096 vs. 0.149 ± 0.098, P=0.493, **Supplementary Table 3**), CV of heart, liver and spinal cord in the AI-assisted arm, was significantly reduced by more than 50% compared with that in the manual contouring arm (all P< 0.05, **Figure 4B**).

**Table 2:** Two methods for delineating OAR volume (cm^3^).

Position	Radiation oncologists	AI-assisted delineation	Difference	*P*
**Left lung**	1357.46 ± 450.12	1321.75 ± 453.27	35.78	<0.001
**Right lung**	1549.31 ± 475.09	1518.07 ± 469.15	31.24	<0.001
**Esophagus**	36.30 ± 9.81	32.77 ± 9.79	3.52	<0.001
**Heart**	637.15 ± 119.98	639.88 ± 123.68	-2.73	0.664
**Liver**	1372.63 ± 354.06	1345.02 ± 350.97	27.61	<0.001
**Cord**	45.52 ± 12.72	40.31 ± 11.63	5.21	<0.001

### Efficiency analysis

**Figure 5:**
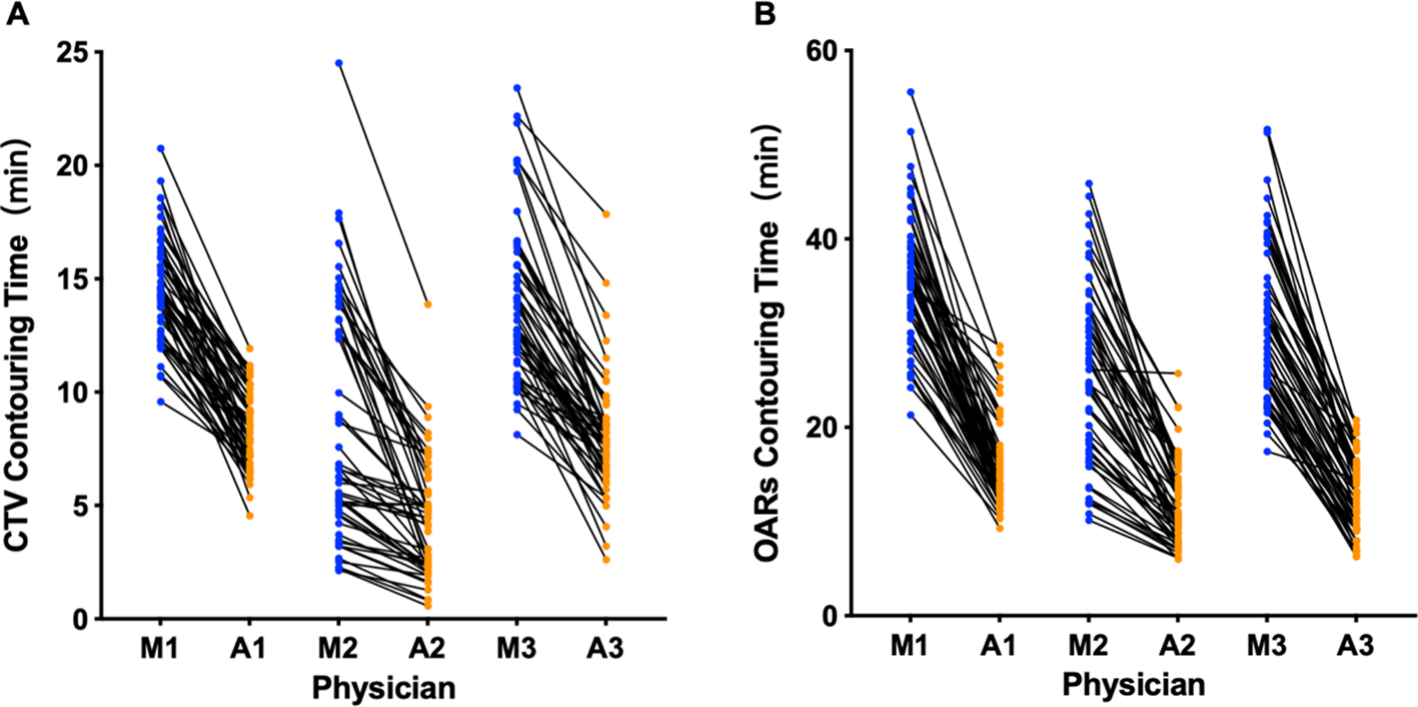
Time for manual and AI-assisted CTV **(A)** and OARs **(B)** delineation. M, manual contour; A, AI-assisted delineation. The numbers represent different physicians.

As illustrated in **Figure 5**, the average time for CTV delineation in the AI-assisted arm was 7.05 ± 1.55 minutes, and the average time for delineating all OARs was 13.44 ± 2.84 minutes. The average time to run the AI model was 56 seconds and 3.8 minutes, respectively. In contrast, fully manual contours by junior radiation oncologists took an average of 12.39 ± 2.28 minutes for CTV delineation and 30.69 ± 6.18 minutes for OARs delineation. Taken together, we observed a significant improvement in CTV and OARs contouring efficiency by approximately 43.2% (5.34 ± 1.75 minutes) and 56.2% (17.73 ± 4.49 minutes), respectively (both P< 0.001). **Supplementary Table 4** shows the time taken to delineate CTV and OARs in different centers. Compared with the manual contour, the CTV shorten time of the three centers is 5.09min, 5.55min, and 7.31min, respectively, and the OARs shorten time is 15.77min, 20.94min, and 26.87min, respectively.

## Discussion

The PORT-C study from the Cancer Hospital of the Chinese Academy of Medical Sciences confirmed that postoperative adjuvant radiotherapy did not improve DFS in patients with stage IIA-N2 NSCLC who underwent complete resection and received 4 cycles of platinum-containing dual-drug adjuvant chemotherapy. The Lung ART study, designed to assess whether patients with non-small cell lung cancer benefit from postoperative radiotherapy after surgical resection, has failed to demonstrate a significant survival benefit for patients with non-small cell lung cancer who receive postoperative radiotherapy compared to those who do not. In view of the high level of evidence in the two phase III randomized controlled studies, postoperative radiotherapy is no longer recommended as a routine recommendation in the treatment guidelines. However, radiotherapy can still be selected for patients with high risk factors such as clinical N2, T3-4, proximal incisal margin of bronchi, extravasal lymph node invasion, positive mediastinal lymph nodes at multiple stations, insufficient number of lymph nodes dissection, and high proportion of metastasis (23, 24).

Accurately delineating CTV for PORT is a critical component of radiation planning, and the quality of CTV delineation PORT basically hinges on the level of clinical expertise possessed by radiation oncologists performing the task. Due to the shortage of medical facilities and workforce in some developing countries, there can an obvious discrepancy in the expertise and clinical skills among physicians from various regions (23–27). Since the globally coronavirus disease 2019 (COVID-19) pandemic, AI-based technology has been paid close attention and promoted more widely in the field of medicine (28–30). For patients with lung cancer, accurate radiation target delineations can result in lower normal organ toxicities and improved long-term survival (31, 32). To the best of our knowledge, this study is the first prospective, multi-center trial to examine the clinical usefulness and practical value of DDCNN model in the automatic delineation of CTV and multiple OARs for lung cancer patients undergoing PORT. Our findings demonstrated that the implementation of AI-assisted technology could lead to significantly improved contouring accuracy, greater interobserver consistency, as well as considerable time savings.

The most commonly used index in the accuracy analysis is the DSC value (33). Our study results showed that, compared with fully manual delineation, the contouring accuracy for CTV of the AI-assisted delineation arm was improved by 5% (0.745 vs. 0.709), relatively similar to the DSC value in mostly prior studies but slightly lower than that in the research focusing on nasopharyngeal cancer (34). The possible reasons include: 1) PORT targets for lung cancer includes bronchial stump and high-risk lymph node area. Determining the residual area after surgery depends on the changes in CT values and window width and level. Unlike patients with nasopharyngeal carcinoma receiving radical radiotherapy, it is difficult to determine the range of delineation for target volumes during PORT for lung cancer patients, since the primary tumor has been removed. 2) Postoperative anatomical changes, including unclear soft tissue boundaries and disorganized organ structures resulting from different lung lobectomy, may affect the learning and construction of AI automatic segmentation models to a certain extent. Therefore, we believe that the introduction of multi-radiomics into AI model training, integrating CT with magnetic resonance imaging and positron emission tomography, on the basis of adaptive postoperative anatomical partition atlas, will further optimize the performance of the AI contouring model.

Moreover, our findings indicated AI-assisted delineation improved the accuracy of OARs contouring by approximately 1%-5%. One possibility that the improvement of OARS is not as large as that of CTV is that the DSC of the target region contoured by manual method are all generally than 0.9, except for the esophagus and spinal cord, and thus the degree of improvement is not obvious. Both the esophagus and spinal cord are tubular organs, and the target area of volumes is small, which is easy to produce DSC difference. In addition, we introduced HD and MDA values to further compare the contouring accuracy by comparing the furthest and average distances of two volumes, and determined the greater accuracy with the AI-assisted method.

After conducting inter-observer consistency evaluation, we observed that the AI-assisted delineation yielded smaller deviations compared to the manual approach. Inter-observer variability in delineating target volumes has been deemed as a major source of uncertainty during the radiotherapy planning (35). Prior studies indicated that even among expert radiation oncologists, significant inter-clinician deviations could be observed during PORT for patients with lung cancer (36). Compared with manual contour, our results found that the CV of the AI-assisted group did not change much in the CTV and lung, which may be due to 1) The delineation of CTV is related to the physician experience. The primary radiation oncologists made more modifications on the target area automatically generated by AI, so there was no significant difference between the two groups; 2) the delineation of delineation was not a traditional manual contour boundary, could be finely modified according to the CT density point selection range, narrowing the gap. 3) Because CV is a statistical index, it is obtained through calculation, and small operations may be ignored during calculation, resulting in insignificant differences. Nevertheless, we found that the AI-assisted group performed well in the delineation of the heart, liver and spinal cord contours, highlighting the significantly improved consistency across junior radiation oncologists and superior clinical usefulness with the assistance of AI auto-segmentation technology.

Furthermore, efficiency gains are the key impetus for promoting the translational application of AI tools in the field of clinical medicine (37). Our study confirms the previous findings that AI-assisted methods could significantly shorten the contouring time for both CTV and OARs (13, 38).

The AI-assisted delineation model has different effects on different levels of hospitals and has certain sociological benefits. For cancer hospitals, the time can be clearly sketched, the efficiency can be improved, and doctors can have more time for diagnosis and treatment. At the same time, it can effectively make up for the lack of technology and experience in primary hospitals, provide more accurate radiotherapy programs, and let primary doctors have a reference standard for learning and comparison. It provides strong evidence for promoting the popularization and application of AI technology in hospitals at different levels. As shown in **Supplementary Figure 1**, the comparison among different centers shows that the manual delineation of LH Hospital is less accurate and takes longer than that of the other two hospitals, but after the assistance of AI, the improvement is the largest. The reason may be that the doctors in lower-level hospitals are not proficient in the anatomy and treatment principles, and there are not many opportunities to delineate the target area in daily work. AI-assisted delineation effectively helps clinicians complete the definition of the target area, significantly shortening the gap with other centers. However, due to the few enrolled patients, the results may not be completely objective.

In the past, the time required for manually delineating primarily depends on 3 factors: clear visualization of the target volume boundaries, in-depth knowledge of anatomical structures and lymph node regions, and comprehensive understanding of the regions at high-risk of failure (13). Currently, the apparent strengths of using AI-assisted model are that it not only facilitates the tissue boundary visualization and radiation target identification, but also provides physicians with instructive contouring optimization, eliminating the overloaded need for manual delineation slice by slice, resulting in a noteworthy improvement in overall working efficiency.

We will standardize the collected clinical information and images of patients, correlate them, and store them as the standard image database of postoperative radiotherapy for lung cancer. The target area of lung cancer radiotherapy mainly relies on the mediastinal lymph node drainage area to be delineated, so we split the existing CTV model and combined with IASLC standard made the following modifications to the common lymph node drainage areas 2, 4, 7 and 10:1) Set the area 2 to start from the level of the lung apex; 2) 2L and 4L inner sections are sketched to the middle line of the trachea, 2R and 4R inner sections are sketched to the left margin of the trachea, allowing overlap; 3) The lower boundary of zone 7 was sketched to 2cm below the carinae, which was connected with zone 8. 4) Zone 10 was sketched according to the lung window, including the main bronchial tree. 100 patients with PORT in the database were selected for zoning sketching, and the AI model was optimized and tested. The results show that the DSC value of the training set can reach 0.85, but the DSC value of the test set is nearly 0.65. The partition model provides a good idea, but it still needs to be further optimized, which is the focus of the next step.

This study has certain limitations. First, given that this trial was conducted in the context of COVID-19, the number of patients presenting to the doctor and receiving PORT decreased significantly, which negatively affected the patient recruitment and sample size of this study. Meanwhile, because the results of the Lung-ART and PORT-C trials were not published until after the start of our trial, the indication for PORT was further restricted, which also postponed the patient enrollment in this study. Besides, AI contouring model relies heavily on the quality and quantity of input data, making it difficult to effectively segment patient images with complex tumor morphology or unclear postoperative anatomical structure, which might lead to the heterogenous and instable performance of AI-assisted model. This study focuses on the application accuracy of AI models in different centers, but does not carry out subfamily analysis of CTV according to left and right position and N stage. After the multi-center data were grouped again, the number of patients in each group was too small, and the experimental results had limitations. In the next step, the number of patients should be added for grouping discussion.

Taken together, our study demonstrated that the AI-assisted delineation model could greatly improve the contouring efficiency of clinicians, reduce the time consumed by manual work, improve the accuracy of both CTV and OARs delineation, narrow the differences in expertise among different radiation oncologists from various medical institutions, and therefore promote the development of intelligent radiation oncology.

## Data Availability

The original contributions presented in the study are included in the article/**Supplementary Material**. Further inquiries can be directed to the corresponding author.
