# Supplementary material for: Artificial intelligence-assisted delineation for postoperative radiotherapy in patients with lung cancer: a prospective, multi-center, cohort study
Source: Front Oncol. 2024 Oct 22;14:1388297. doi: 10.3389/fonc.2024.1388297 (PMC11579590; doi:10.3389/fonc.2024.1388297)
Supplement: Supplementary file 1 [file DataSheet1.docx]

**Supplementary materials:**

**Table 1. Accuracy of CTV delineated by two methods in different centers**

|  | **Center** | **Radiation oncologists** | **AI-assisted delineation** | **Median difference** |
| --- | --- | --- | --- | --- |
| **HD** | **CAMS**  **BZ**  **LH** | 21.92 (16.61, 32.45)  13.37 (11.68, 18.18)  25.78 (21.41, 30.68) | 21.71 (17.01, 30.03)  10.42 (7.92, 13.67)  15.28 (11.61, 27.21) | 1.08 (0.20-1.91)  4.24 (1.02-0.7.92)  9.79 (5.16-13.63) |
| **MDA** | **CAMS**  **BZ**  **LH** | 2.99 (2.36, 4.03)  1.99 (1.86, 2.53)  4.09 (3.51, 5.66) | 2.73 (2.18, 3.68)  1.31 (1.02, 2.34)  2.01 (1.61, 2.91) | 0.24 (0.15-0.35)  0.68 (0.20-1.15)  2.00 (1.42-2.61) |
| **DSC** | **CAMS**  **BZ**  **LH** | 0.711 (0.669, 0.757)  0.692 (0.642, 0.756)  0.639 (0.598, 0.675) | 0.744 (0.713, 0.781)  0.774 (0.720, 0.819)  0.753 (0.726, 0.792) | 0.027 (0.019-0.035)  0.066 (0.035-0.099)  0.117 (0.088-0.150) |

All *P* values < 0.05, calculated by Hodges Lyman estimation.

***Abbreviations***: HD, Hausdorff distance(mm); MDA, mean distance to agreement(mm); DSC, dice coefficient; CI, confidence interval.

**Table 2. OARs accuracy analysis**

| **Position** |  | **Radiation oncologists** | **AI-assisted delineation** | | **Median difference** |
| --- | --- | --- | --- | --- | --- |
| **Left lung** | **HD**  **MDA**  **DSC** | 16.48 (12.23, 24.28)  0.60 (0.49, 0.81)  0.974 (0.97, 0.98) | 12.65 (8.28, 17.03)  0.28 (0.14, 0.57)  0.988 (0.98, 0.99) | 4.52 (3.30-5.70)  0.37 (0.32-0.39)  0.015 (0.013-0.017) | |
| **Right lung** | **HD**  **MDA**  **DSC** | 18.11 (14.10, 21.74)  0.74 (0.47, 0.89)  0.975 (0.97, 0.99) | 12.50 (9.20, 17.35)  0.36 (0.18, 0.71)  0.987 (0.97, 0.99) | 4.46 (3.47-5.48)  0.36 (0.28-0.42)  0.008 (0.006-0.010) | |
| **Esophagus** | **HD**  **MDA**  **DSC** | 12.37 (9.21, 18.21)  1.18 (0.98, 1.53)  0.767 (0.73, 0.80) | 10.12 (7.57, 14.83)  1.05 (0.84, 1.28)  0.780 (0.74, 0.82) | 2.31 (1.51-3.28)  0.18 (0.13-0.25)  0.019 (0.010-0.028) | |
| **Heart** | **HD**  **MDA**  **DSC** | 10.99 (9.13, 15.00)  1.61 (1.37, 2.21)  0.931 (0.91, 0.94) | 8.75 (6.29, 11.27)  0.65 (0.32, 1.11)  0.972 (0.95, 0.99) | 2.63 (2.02-3.26)  0.98 (0.86-1.09)  0.041 (0.036-0.046) | |
| **Liver** | **HD**  **MDA**  **DSC** | 16.69 (12.88, 20.79)  1.46 (1.21, 1.70)  0.946 (0.94, 0.95) | 12.70 (9.54, 17.79)  0.37 (0.24, 0.93)  0.986 (0.97, 0.99) | 2.91 (2.16-3.70)  0.94 (0.86-1.02)  0.036 (0.033-0.038) | |
| **Cord** | **HD**  **MDA**  **DSC** | 7.19 (5.21, 15.03)  0.98 (0.80, 1.28)  0.796 (0.75, 0.83) | 6.01 (4.58, 10.01)  0.82 (0.46, 1.22)  0.826 (0.76, 0.90) | 1.21 (0.58-2.35)  0.20 (0.17-0.28)  0.039 (0.023-0.054) | |

All *P* values < 0.05, calculated by Hodges Lyman estimation.

***Abbreviations***: HD, Hausdorff distance(mm); MDA, mean distance to agreement(mm); DSC, dice coefficient; CI, confidence interval.

| **Position** | **Radiation oncologists** | **AI-assisted delineation** | ***P*** |
| --- | --- | --- | --- |
| **CTV**  **Left lung**  **Right lung**  **Esophagus**  **Heart**  **Liver**  **Cord** | 0.122 (0.071, 0.200)  0.004 (0.002, 0.008)  0.007 (0.003, 0.016)  0.137 (0.081, 0.194)  0.045 (0.022, 0.063)  0.028 (0.019, 0.036)  0.192 (0.119, 0.276) | 0.113 (0.063, 0.207)  0.003 (0.005, 0.007)  0.006 (0.003, 0.011)  0.128 (0.055, 0.172)  0.013 (0.005, 0.029)  0.005 (0.003, 0.009)  0.068 (0.023, 0.130) | 0.503  0.669  0.045  0.156  <0.001  <0.001  <0.001 |

**Table 3. Two methods for CV values of CTV and OARs**

**Table 4. Time of CTV and OARs delineation by different centers using two methods. (min)**

| **Position** | **Center** | **Radiation oncologists** | **AI-assisted delineation** | **Difference value** |
| --- | --- | --- | --- | --- |
| **CTV** | **CAMS**  **BZ**  **LH** | 11.94±2.21  13.28±0.77  15.50±0.76 | 6.84±1.62  7.73±0.26  8.19±1.06 | 5.09±1.74  5.55±0.73  7.31±1.42 |
| **OARs** | **CAMS**  **BZ**  **LH** | 28.67±4.65  37.08±1.74  45.52±1.78 | 12.90±2.83  16.14±1.01  15.64±0.99 | 15.77±3.08  20.93±2.33  25.88±2.08 |

All *P* values < 0.05

**
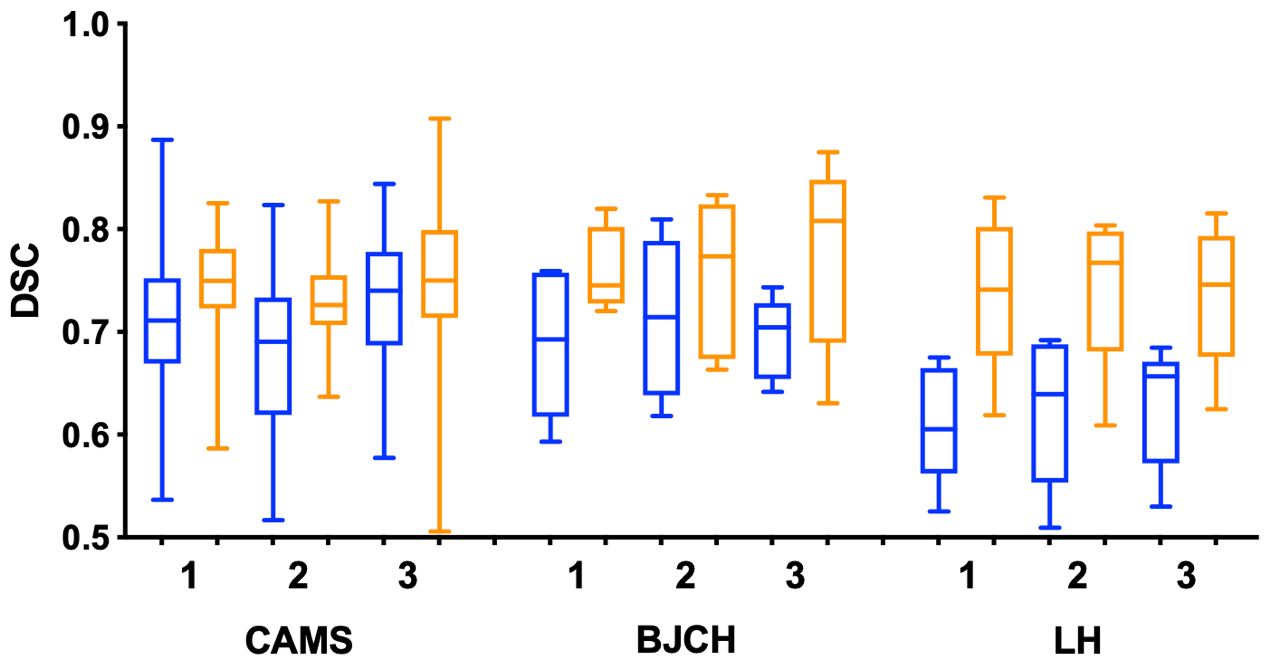
**

**Figure 1.** Comparison of DSC of CTV in different centers.

***Abbreviations***: CAMS, Cancer Hospital of Chinese Academy of Medical Sciences; BJCH, Beijing Cancer Hospital and Institute; LH, Beijing Luhe Hospital of Capital Medical University. The numbers represent different physicians.
